# Proteomic Analysis of Etiolated Juvenile Tetraploid *Robinia pseudoacacia* Branches during Different Cutting Periods

**DOI:** 10.3390/ijms15046674

**Published:** 2014-04-21

**Authors:** Nan Lu, Zhaohe Xu, Bingnan Meng, Yuhan Sun, Jiangtao Zhang, Shaoming Wang, Yun Li

**Affiliations:** 1 National Engineering Laboratory for Tree Breeding, Key Laboratory of Genetics and Breeding in Forest Trees and Ornamental Plants, Ministry of Education, College of Biological Sciences and Technology, Beijing Forestry University, Beijing 100083, China; E-Mails: ln890110@gmail.com (N.L.); mengbn80@gmail.com (B.M.); syh831008@gmail.com (Y.S.); 2 Key Laboratory for Silviculture and Conservation, Ministry of Education, Beijing Forestry University, Beijing 100083, China; E-Mail: xuzhaohecn@gmail.com; 3 Beijing Municipal Bureau of Landscaping, Beijing 100029, China; 4 Henan Academy of Forestry Science, Zhengzhou 450008, Henan, China; E-Mail: zhjt5966@gmail.com; 5 The State-Owned Luoning County Lv Cun Forest Farm, Luoning 471711, Henan, China; E-Mail: wangshaoming1969@gmail.com

**Keywords:** tetraploid *Robinia pseudoacacia*, etiolated juvenile branch, cutting, proteomics

## Abstract

The propagation of hard-branch cuttings of tetraploid *Robinia pseudoacacia* (black locust) is restricted by the low rooting rate; however, etiolated juvenile tetraploid black locust branches result in a significantly higher rooting rate of cuttings compared with non-etiolated juvenile tetraploid branches. To identify proteins that influence the juvenile tetraploid branch rooting process, two-dimensional electrophoresis (2-DE) and matrix-assisted laser desorption/ionization time-of-flight/time-of-flight mass spectra (MALDI-TOF/TOF-MS) were used to analyze proteomic differences in the phloem of tetraploid *R. pseudoacacia* etiolated and non-etiolated juvenile branches during different cutting periods. A total of 58 protein spots differed in expression level, and 16 protein spots were only expressed in etiolated branches or non-etiolated ones. A total of 40 highly expressed protein spots were identified by mass spectrometry, 14 of which were accurately retrieved. They include nucleoglucoprotein metabolic proteins, signaling proteins, lignin synthesis proteins and phyllochlorin. These results help to reveal the mechanism of juvenile tetraploid *R. pseudoacacia* etiolated branch rooting and provide a valuable reference for the improvement of tetraploid *R. pseudoacacia* cutting techniques.

## 1. Introduction

Compared to normal diploid *Robinia pseudoacacia*, tetraploid *R. pseudoacacia* clones have a significantly higher yield with large leaves and a high leaf protein content; moreover, they are polyanthous and long blossoming and suitable for feeding and beekeeping. Recent studies have shown that tetraploid *R. pseudoacacia* are fast-growing and able to tolerate harsh environments, including salt and drought; thus, they are widely planted for soil and water conservation, environmental improvement, wind attenuation and sand fixation. Additionally, they have good ecological and economic benefits and can be used in the development of stockbreeding on plains and mountains, among other aspects [1,2].

Because of the high seedling abortion rate and the desire to maintain valuable clonal traits, efficient asexual reproduction methods for tetraploid *R. pseudoacacia* propagation are imperative. Traditional asexual reproduction methods mainly include root cutting, branch cutting, tissue culture and grafting. In China, many labs have successfully established a tissue culture system for tetraploid *R. pseudoacacia*. By using root segments after sand storage treatment in winter, Dong [3] obtained clonal seedlings with a relatively high survival ratio. As for grafting, Shang [4] obtained a 90% survival ratio through cleft grafts.

However, these methods have their limitations in terms of production. Root cuttings hurt the root system of mother trees, and the number of seedlings is restricted by mother trees. Tissue culture requires a significant monetary investment and professional techniques; thus, the production cost is very high. Grafting has a longer propagation cycle, usually requiring at least one year to culture rootstocks, and because of the higher growth rate of scions it easily suffers wind-breakage and out-of-line forestems during the graft seedling growing period.

Compared with other asexual propagation methods, branch cutting has several advantages, including being a rich source of branches, harmless to the mother tree, and requiring little monetary investment. By establishing a cutting orchard, we can obtain a number of cutting slips outside of the growing season without hurting the mother tree [1,2]. However, the rooting rate of tetraploid *R. pseudoacacia* hard-branch cuttings using traditional techniques is only 4% [5]. Improvements to cutting technology could improve the rooting rate dramatically [6]. Taking advantage of etiolated (causing new buds or juvenile branches to develop without chlorophyll by growing without exposure to sunlight before cutting from the mother tree) juvenile branches as cutting materials is a common way to improve cutting rooting rates and has beneficial effects on the rooting of avocado [7], apples [8], maples [9] and other plants. Etiolation not only restrains the production of materials that hinder the rooting process, enhancing the activity of endogenous hormones, it also slows down the lignification of cut slips [10]. In a previous study on the physiology and biochemistry of juvenile tetraploid *R. pseudoacacia* branches, we found that regulation of the level of endogenous hormones and oxidase activity promoted the rooting of etiolated branches [11]; however, this study did not elucidate the mechanism of etiolated juvenile branch rooting.

Proteomics is an effective way to study the mechanism of rooting. Using proteomics, Konishi *et al.* [12] found that increases in fructose-bisphosphate aldolase activity may play an important role in gibberellic acid-induced root growth. Proteomic studies of mutated *Arabidopsis thaliana* also helped to elucidate the rooting mechanism [13]. To further study the mechanism of tetraploid *R. pseudoacacia* juvenile branch rooting, we sought to identify proteins that influence the rooting process.

We hypothesize that perhaps the rooting improvement of etiolated juvenile branch was due to the regulation of the expression level of some rooting associated proteins or there are some proteins that promote the rooting of etiolated juvenile branch exist. Therefore, it is necessary to determine differences between proteins in etiolated and non-etiolated juvenile branch during the rooting periods. Since there was no previous study examining proteome levels during the tetraploid *R. pseudoacacia* etiolated juvenile branch cutting period, and no data on this topic are currently available in the literature. Therefore, this study can help us to better understand the mechanism of juvenile branch rooting and provide a reference for improving the cutting technology for tetraploid *R. pseudoacacia* juvenile branches.

## 2. Results

### 2.1. Comparative Protein Profiles of Non-Etiolated and Etiolated Juvenile Branch Cuttings during Different Rooting Periods

All proteins were detected on Coomassie brilliant blue (CBB)-stained gels. The identified spots corresponded to proteins expressed in different periods. Spots of non-etiolated samples were chosen as controls. On day 0, four proteins were up-regulated, while seven were down-regulated, four spots were not present and one spot was only present in the etiolated samples, compared with non-etiolated samples. On day 3, there were nine spots up-regulated spots, six spots down-regulated spots, three protein spots were not present and there were two unique spots. On day 9, five spots were up-regulated, and seven spots changed in the opposite way, five spots disappeared and one spots only expressed in etiolated samples. In total, 38 protein spots were differentially expressed and 16 spots were only expressed during certain periods in non-etiolated and etiolated branches.

To accurately assess the proteomic changes, spot volume differences of more than two-fold between two identical spots were defined as significant. Significantly differentially expressed spots were subjected to matrix-assisted laser desorption/ionization time-of-flight/time-of-flight mass spectra (MALDI-TOF/TOF-MS) to confirm their identity and function.

### 2.2. Matrix-Assisted Laser Desorption/Ionization Time-of-Flight/Time-of-Flight Mass Spectra (MALDI-TOF/TOF-MS) Identification of Etiolation-Responsive Proteins

**Figure 1.**
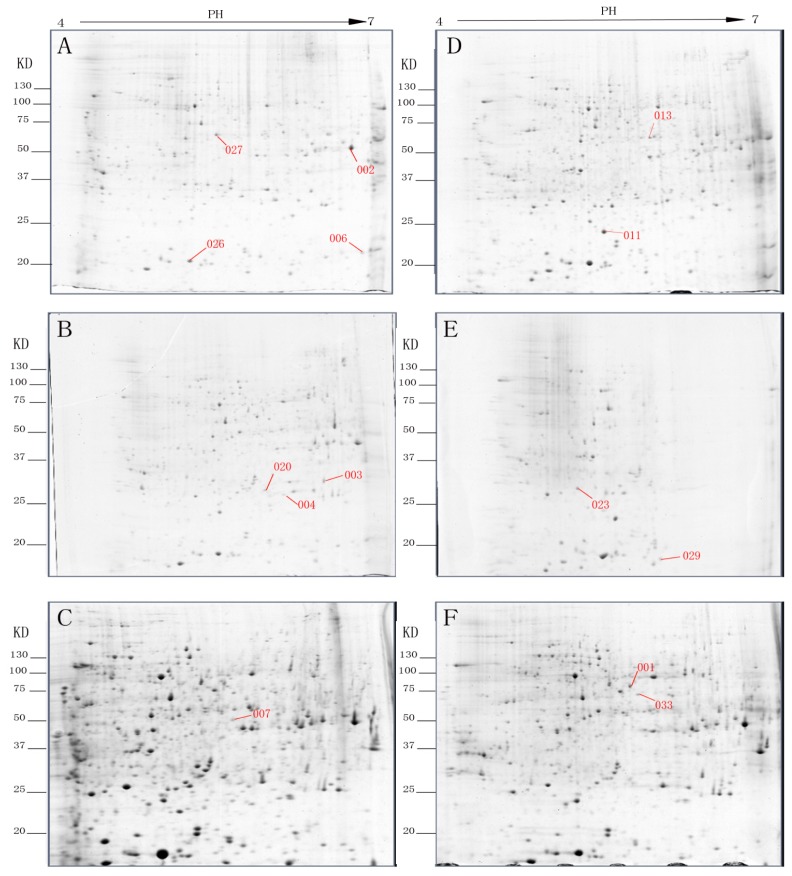
Representative two-dimensional electrophoresis (2-DE) gels of rooting of etiolated juvenile tetraploid *Robinia pseudoacacia* branches after (**A**) day 0; (**B**) day 3; and (**C**) day 9. Representative 2-DE gels of rooting of non-etiolated juvenile tetraploid *Robinia pseudoacacia* branches after (**D**) day 0; (**E**) day 3; and (**F**) day 9.

Forty proteins from the differentially expressed spots between the non-etiolated and etiolated samples were arbitrarily selected from the gels and subjected to MALDI-TOF/TOF-MS. Thirty-three proteins were successfully identified. Among the identified proteins, 14 were identified, while 19 were hypothetical proteins or predicted proteins (Figure 1). The identified proteins are listed in Table 1. The fourteen proteins include ribosomal proteins, metabolic proteins, lignin synthesis proteins and chlorophyll proteins.

The identified spots (Figure 1) correspond to proteins related to energy, carbohydrate metabolism and photosynthesis. Compared to the non-etiolated branches, seven proteins were up-regulated (spots 002, 003, 004, 006, 020, 026 and 027) and two were down-regulated (spots 001 and 029). One spot was only expressed in etiolated branches (spot 007) while the rest were not present (spots 011, 013, 023 and 033) in the etiolated branches.

Glycine-rich protein (GRP, spot 033), an important structural protein present in plant cell walls [14], and Se-wap41, a protein involved in cell wall polysaccharide synthesis [15], were not expressed in the etiolated branches. This indicates that etiolation may interfere with cell wall formation.

Methylmalonate-semialdehyde dehydrogenase (MM-ALDH, spot 002), enolase (spot 007), ribulose-1,5-bisphosphate carboxylase (Rubisco) large subunit (spot 011), Mat K (spot 013) and heat shock protein (HSP, spot 027) are involved in plant stress responses. Their expression suggests that etiolation improves the survivability of juvenile branches under stress.

## 3. Discussion

Proteins, as the products of the comprehensive expression of functional genes, directly reflect biological function and impact the growth and development of plants. Through a proteomic comparison of non-etiolated and etiolated cuttings during different rooting periods, we sought to better understand the rooting mechanism of tetraploid *R. pseudoacacia* branches and the response of individual proteins on the basis of their function.

### 3.1. Cell Wall Metabolism and Remodeling

Plant morphogenesis includes mechanisms to control the balance between cell division, cell expansion and cell adhesion [16–18]. In our study, we found two proteins (spots 033 and 023, identified as GRP and Se-wap41, respectively) associated with metabolism and the remodeling of cell walls.

GRP (spot 033), an important structural protein in plant cell walls, was not present in etiolated specimens and was found to be down-regulated. GRP in the cell wall has a structural function and plays a role in bracing and adhesion and in the distribution of other components of the cell wall [19]. GRP also participates in the reparative process of xylem conduit primary walls after passive stretching [20].

Se-wap41 was only expressed in non-etiolated branches. Se-wap41 is a 41-kDa wall-associated protein that was reported to label plasmodesmata in the Golgi and class I reversibly glycosylated polypeptide (RGP) [15]. RGPs are thought to be involved in cell wall polysaccharide synthesis [21–25]. Using immunogold labeling, Dhugga *et al.* [26] showed that RGP1 was specifically localized to Golgi stacks, where it is likely involved in xyloglucan biosynthesis [27].

According to Ludwig *et al.* [28], maceration of cell walls where adventitious root primordial formation was in progress was better for the formation of adventitious roots. In a study of adventitious root development in *Pinus contorta*, Brinker *et al.* [29] found that during the first 3 days after auxin treatment, genes with the potential to be active in cell wall synthesis undergo down-regulation, and, at the same time, genes involved in weakening cell walls and cell adhesion were up-regulated.

### 3.2. Proteins Involved in Carbohydrate Metabolism

Respiration is critical to metabolism in higher plants: it releases the energy stored in carbon-based compounds, providing it for the organism and for physiological activity.

Fructokinase (FRK) (spot 026) is an important metabolic signaling enzyme and key enzyme in sucrose decomposition [30]. Sucrose can be directly stored or transformed into hexose for storage and for respiratory metabolism. Sucrose is converted to glucose and fructose by invertase and then phosphorylated by FRK and hexokinase [31,32]. Sucrose, glucose and fructose greatly stimulate the induction of adventitious roots [33]. Enolase (spot 007) has a role in the Embden-Meyerhof-Parnas pathway (EMP), which responds to many environmental stressors, including salt, drought, cold and hypoxia [34]. The enolase content in maize is increased 1.4 times during stress [20]. Enolase appeared only in etiolated branches in this study (day 9). The EMP pathway provides large amounts of carbohydrates for the Krebs cycle [35], and its product, pyruvic acid, is very active and indirectly participates in the regulation of adventitious root development through various metabolic pathways [36].

Spot 011 (Rubisco large subunit) is the catabolite of Rubisco. Rubisco is a key enzyme in CO_2_ fixation during photosynthesis and is involved in photorespiration in plants [37]. As one of the main organic nitrogen compounds in plants, Rubisco is related to the uptake and utilization of nitrogen [37]. Under stressful conditions, Rubisco is prone to degradation, ultimately inhibiting photosynthesis [38], while low degradation rates for Rubisco are beneficial for the formation of adventitious roots [36]. Spot 011 appeared only in non-etiolated plants (day 0).

### 3.3. Proteins Involved in Metabolism

4-Coumarate-CoA ligase (4CL) (spot 029) is a key lignin synthesis enzyme, the last enzyme in phenylpropyl derivative metabolism and an important enzyme in the divergent synthetic pathway of natural phenylpropyl derivatives [39]. 4CL activity is associated with the plant lignin content and with the contents of cinnamic aldehyde and S residues in lignin molecules. The lignin content should decrease when 4CL activity is restricted [40–42]. Cho [43] found that indole-3-butyric acid (IBA)-treated *Cinnamomum kanehirae* cuttings had lower amounts of lignin and exhibited greater induction of adventitious roots. 4CL only appeared during adventitious root primordium formation (day 3) in non-etiolated branches. We speculate that through a decrease in lignin, etiolated branches produce more root primordia during the formation of adventitious roots.

MM-ALDH (spot 002) is encoded by a single gene, ALDH6B2, in *A. thaliana*. MM-ALDH is associated with the degradation of valine to propionyl coenzyme A, but this enzyme is not well studied and its expression has not been clarified [44]. Currently, studies of ALDH have mainly focused on abiotic stress [45,46]. Gao *et al.* [47] found that genes from eight ALDH gene families (2, 3, 5, 7, 10, 11, 18 and 22) responded to drought and salt stress. Chen *et al.* [48] found that BADH (*ALDH* gene family 10) was able to oxidize betaine aldehyde to betaine, which maintains the osmotic balance of plant cells and enhances plant stress resistance. MM-ALDH was up-regulated, especially on day 0, and may be the result of stress during the early cutting period.

### 3.4. Transcription, Translation and Signal Transduction

Mat K (spot 013) participates in shearing the introns of type II chloroplast RNA transcripts, thereby influencing the expression of regulatory genes [49]. Jia *et al.* [50] speculated that when Mat K and other chloroplast genes are up-regulated, metabolic disorders of the chloroplast result, leading to bud death. They conjectured that programmed cell death may be a physiological phenomenon commonly occurring when plants abort buds, age and are under stress. Mat K was only present during the initial period (day 0) in non-etiolated branches; in comparison, it was weakly expressed in etiolated ones. The higher chlorophyll content in non-etiolated branches may be a reason for this differential expression pattern or it may be due to stress during the cutting process.

Ribosomal protein S4 (spot 006) is a ribosomal protein that functions in protein synthesis. In mammals, ribosomal protein S4 is located at the junction of the 40S and 60S subunits and interacts with initiation factor Eif-3 [51]. Ribosomal protein S4 may be related to the interaction between mRNAs and ribosomes [51]. We know little about the relationships between ribosomal protein S4 and the structure-function of ribosomes in plants. Ribosomal protein S4 has only been cloned in potato and cotton. The up-regulation of ribosomal protein S4 may increase mRNA expression, thereby promoting the formation of adventitious roots.

Spot 001 corresponds to an RNA-binding protein belonging to a large protein family containing DNA and RNA recognition sequences and involved in regulating the alternative splicing of RNA. They also play an important role in regulating transcriptional processing, translation and RNA metabolism [52,53]. Spot 001 was highly expressed in non-etiolated branches and may be associated with the strong metabolic activity of non-etiolated branches. The 54-kDa signal recognition particle (SRP, spot 003) is a chloroplast signal recognition protein. The SRP plays an important role in the transport of endoplasmic reticulum-secreted proteins to the cell membrane. It contains SRP 7S RNA and six proteins, including a 54-kDa protein signal receiver (SRP54) identifying the original polypeptide signal sequence and a 19-kDa signal receiving protein that adheres to the SPR 7S RNA directly, which is important in the recognition and binding of the SPR54 particle [54]. Both secreted proteins and membrane proteins have a target receptor signal sequence. The SRP identifies the signal sequence in the initial polypeptide sequence, binding and extending the polypeptide sequence [55–57].

### 3.5. Protein Metabolism

HSP (spot 006) has been widely studied, especially for its functions in stress. HSP is normally expressed in the cytoplasm of cell bodies and axons, where it binds to and stabilizes microtubules, promoting the solubility of proteins and protein binding to microtubules [58]. In our study, HSP was up-regulated in etiolated branches. Up-regulated HSP helps maintain the functions of many proteins, sustaining normal physiological function.

Actin is an important protein generally present in eukaryotic cells and involved in many activities. Actin is a single polypeptide chain of globular proteins (G-actin) consisting of 375–377 amino acids. Actin is an important component of the plant cytoskeleton and microfilaments and contributes to cell elongation, probably through interactions with cortical microtubules [59]; it also maintains polar auxin transport [60]. Spots 004 and 020 were identified as actin. In our study, we found that on days 0 and 3 the expression of the two actin proteins were not obviously different between the etiolated samples and controls, but on day 9 the expression in the controls was very low. Day 9 marks the period of adventitious root elongation, and the weak expression of actin has a negative influence on the elongation of adventitious roots.

## 4. Experimental Section

### 4.1. Chemicals and Reagents

Tris, octylphenoxypolyethoxyethanol (NP-40), urea, sulfourea, 3-[(3-Cholamidopropyl)dimethylammonio]-1-propanesulfonate (CHAPS), sodium dodecyl sulphate (SDS), acrylamide, *N*,*N*′-methylenebisacrylamide, ammonium persulfate, *N*,*N*,*N*′,*N*′-tetramethylethylene diamine, CBB G-250 and trichloroacetic acid were obtained from Amresco (Solon, OH, USA); phenylmethylsulfonyl fluoride was obtained from Sigma (St. Louis, MO, USA); immobilized pH gradient strips (IPG strips, 17 cm, pH 4–7) and iodoacetamide were obtained from Bio-Rad Laboratories (Hercules, CA, USA); acetone, glycerol, phosphoric acid, carbinol and alcohol (analytical reagents) were obtained from manufacturers in China. All water used in this experiment was Milli-Q hyperpure water (Millipore, Billerica, MA, USA).

### 4.2. Plant Materials

Non-etiolated and etiolated juvenile branch cuttings of tetraploid *R. pseudoacacia* K4 clones [treated with 2000 mg/L indole-3-butyric acid (IBA) before cutting] at different stages of rooting [callus induction (day 0), adventitious root primordium formation (day 3), and adventitious root elongation] were collected from Lucun National Forest Farm (Luoning, Henan, China) in the spring. Phloem from a branch 2 cm from the base was stripped using a knife and placed immediately in liquid nitrogen.

### 4.3. Protein Extraction

Total protein extracts were prepared according to the method of Wang *et al.* [10] with minor modifications. A total of 2 g of powdered phloem were suspended in 6 mL of cold extraction solution (10% trichloroacetic acid in acetone containing 0.07% β-mercaptoethanol) and then incubated at −20 °C for 6 h. The samples were then centrifuged at 13,000× *g* for 25 min at 4 °C and the supernatant was discarded. The last step was repeated 2–3 times. The pellet was dried under a vacuum for 10 min at 4 °C and stored at −70 °C.

The dried pellet was dissolved in lysis buffer containing 8 M urea, 2 M thiourea, 4% CHAPS, 60 mM dithiothreitol (DTT) and 0.5% IPG buffer (pH = 4.7), then oscillated on an oscillator for 2 min, incubated at 28 °C in a water bath for 30 min and then centrifuged at 13,000× *g* for 30 min at room temperature. The protein concentration of the supernatant was determined by the Bradford method.

### 4.4. Two-Dimensional Electrophoresis (2-DE)

Two-dimensional gel electrophoresis was performed in accordance with the Bio-Rad handbook. A total of 1.3 mg of sample was loaded onto analytical and preparative gels. For isoelectric focusing, the Bio-Rad Mini-PROTEAN II 2-D system and IPG strips (pH 4–7, 17 cm) were used according to a preliminary experiment. The IPG strips were rehydrated for 12 h in 350 μL of rehydration buffer containing protein.

The gel strips were equilibrated twice for 15 min each with 10 mL of the following equilibration buffers: (i) reducing buffer for 15 min (0.373 M Tris-HCl buffer, pH 8.8, 6 M urea, 20% *v*/*v* glycerol, 2% *w*/*v* SDS and 0.2 g of DTT); and (ii) alkylating buffer for 15 min (0.373 M Tris-HCl buffer, pH 8.8, 6 M urea, 20% *v*/*v* glycerol, 2% *w*/*v* SDS and 2.5 g of iodoacetamide). SDS-PAGE was performed on 12% gels using the PROTEAN II xi cell system (Bio-Rad Laboratories). The gels were run for 0.5 h at 5 mA per gel, then at 20–30 mA per gel until the dye front reached the gel bottom. The proteins were stained with CBB G-250.

### 4.5. Image Acquisition and Data Analysis

Two-dimensional electrophoresis images were obtained using a Scanner GS-800 (Bio-Rad Laboratories) in transmission mode. Image analysis was done using a combination of manual visualization and software calculations with PDQuest Gel Analysis Software (Bio-Rad Laboratories). All 2-DE images were globally analyzed by the software for spots that significantly differed in a comparison of the different rooting periods.

### 4.6. Trypsin Digestion

The CBB-stained spots were excised from the gels and destained for 30 min until the gel was transparent using a destaining solution containing 50% acetonitrile (ACN) and 25 mM ammonium bicarbonate (NH_4_HCO_3_).

The gels were dried for 30 min using the Thermo Savant Speed-Vac system. A total of 3 mL of trypsin solution (final concentration 10 ng/μL dissolved in 25 mM NH_4_HCO_3_) were pipetted onto each dried protein spot and the sample was incubated at 4 °C for 1 h. The supernatant was discarded and the Ep tubes were placed upside down and incubated at 37 °C for 15 h. To extract the peptide fragments from the trypsin digest, 50 μL of 5% (*v*/*v*) trifluoroacetic acid (TFA) were added to the samples and incubated at 40 °C for 1 h. Next, the supernatant was transferred to another clean Ep tube. A total of 50 μL of 50% (*v*/*v*) ACN [containing 2.5% (*v*/*v*) TFA] were added to the gel and the samples were incubated at 30 °C for 1 h. The supernatant was collected and dried in a Speed-Vac system then stored at 4 °C.

### 4.7. Protein Identification by MALDI-TOF/TOF-MS

The digested peptides were desalted and cleaned with ZipTip C18 pipette tips (Millipore Corp., Bedford, MA, USA) before obtaining the mass spectrum of the peptide mixture. All analyses were performed using a Bruker Daltonics Autoflex (Bruker Daltonics, Billerica, MA, USA) operated at 350 nm Nd YAG with an accelerating voltage of 20 kV. The peptide mixture was analyzed using a saturated solution of *R*-*cyano*-4-hydroxycinnamic acid in 50% ACN/0.1% TFA. External calibration was performed with peptides from myoglobin and internal calibration with trypsin autoproteolytic fragments. The samples were analyzed on a MALDI-TOF/TOF-MS 4700 proteomics analyzer (Agilent Technologies, Santa Clara, CA, USA) and the data were analyzed using MASCOT software (Matrix Science, London, UK).

## 5. Conclusions

In our study, tetraploid *R. pseudoacacia* juvenile branches were subjected to etiolation followed by a proteomic analysis of treated and control samples in different rooting periods. A total of fourteen etiolation-responsive proteins were successfully identified (Table 1) by 2-DE and MALDI-TOF/TOF-MS. Proteins are the most direct reflection of plant etiolation responses. Through further analysis, we can determine the possible roles for the fourteen proteins in promoting the rooting of juvenile branches. These differentially expressed proteins should be the focus of future studies. The real factor responsible for the higher rooting rate in etiolated juvenile branches might be found among them. More work needs to be done on these proteins, including comparing them with etiolation-responsive proteins in other species to determine if any of them are shared. In our future work, we plan to compare the expression between etiolated and non-etiolated juvenile tetraploid *R. pseudoacacia* branches at the transcriptome level during different rooting periods, through which we hope to find the genes associated with rooting. If we can confirm the significant proteins and genes and determine the mechanism of the higher rooting rate in juvenile etiolated tetraploid *R. pseudoacacia* branches, it would improve the cutting technology of tetraploid *R. pseudoacacia* trees.

## Figures and Tables

**Table 1.** Protein identification through matrix-assisted laser desorption/ionization time-of-flight/time-of-flight mass spectra (MALDI-TOF/TOF-MS).

Protein ID	Accession No.	Description	Score	SC (%)	Theoretical *M*_r_ (kDa)/pI	Observed *M*_r_ (kDa)/pI
001	gi|116061758	Putative RNA-binding protein	92	28	72.4/8.51	78.3/5.26
002	gi|168009419	Methylmalonate-semialdehyde dehydrogenase (MM-ALDH)	74	17	61.5/8.51	57.1/6.33
003	gi|108864006	Signal recognition particle 54 kDa protein, chloroplast precursor, putative, expressed [*Oryza sativa* (japonica cultivar-group)]	80	19	53.3/9.37	38.8/6.14
004	gi|60650116	Actin [*Pyrus communis*]	106	28	38.4/5.47	28.6/5.91
005	gi|242080859	Hypothetical protein SORBIDRAFT_07g005770 [*Sorghum bicolor*]	82	21	71.8/6.17	65.8/5.43
006	gi|121761863	Ribosomal protein S4 [*Plagiomnium cf. tezukae Wyatt 1808*]	83	12	23.5/10.39	23.88/6.61
007	gi|42521309	Enolase [*Glycine max*]	87	15	47.7/5.31	52.2/5.05
008	gi|152143640	Chloroplast photosynthetic water oxidation complex 33 kDa subunit precursor [*Morus nigra*]	78	16	28.2/5.48	35.2/5.84
009	gi|242080859	Hypothetical protein SORBIDRAFT_07g005770 [*Sorghum bicolor*]	82	21	71.8/6.17	75.4/5.88
010	gi|270306046	Unnamed protein product [*Vitis vinifera*]	98	16	43.2/9.36	50.3/5.43
011	gi|67079128	Ribulose-1,5-bisphosphatecarboxylase/oxygenase large subunit [*Chasmanthium latifolium*]	90	14	25.2/5.82	24.5/5.52
012	gi|255559120	Cytosolic purine 5-nucleotidase, putative [*Ricinus communis*]	82	12	62.7/6.67	72.1/6.22
013	gi|166156335	Maturase K [*Protea neriifolia*]	83	17	60.0/9.51	64.2/5.83
014	gi|147814811	Hypothetical protein [*Vitis vinifera*]	104	25	78.8/6.23	85.7/6.54
015	gi|242081717	Hypothetical protein SORBIDRAFT_07g022905 [*Sorghum bicolor*]	73	8	21.0/5.73	19.2/6.47
016	gi|168044879	Predicted protein [*Physcomitrella patens subsp. patens*]	74	9	38.8/9.45	40.8/5.43
017	gi|13928452	14-3-3 Protein [*Vigna angularis*]	108	21	29.2/4.66	35.2/5.21
018	gi|255559120	Cytosolic purine 5-nucleotidase, putative [*Ricinus communis*]	82	12	62.7/6.67	72.6/6.08
019	gi|224141801	Predicted protein [*Populus trichocarpa*]	84	18	60.9/7.04	68.2/5.92
020	gi|224055984	Actin 1 [*Populus trichocarpa*]	130	26	41.7/5.31	33.7/5.82
021	gi|225448323	Predicted: hypothetical protein [*Vitis vinifera*]	126	28	41.6/5.31	35.2/6.05
022	gi|125563066	Hypothetical protein OsI_30711 [*Oryza sativa Indica Group*]	78	25	88.7/9.13	85.5/5.67
023	gi|162463414	Golgi associated protein se-wap4 [*Zea mays*]	88	18	41.2/5.75	32.8/5.30
024	gi|255554359	Conserved hypothetical protein [*Ricinus communis*]	74	14	60.7/6.12	68.4/5.76
025	gi|225437076	Predicted: hypothetical protein isoform [*Vitis vinifera*]	82	16	63.9/7.18	71.4/6.37
026	gi|194466127	Fructokinase [*Arachis hypogaea*]	76	11	20.1/5.07	20.6/5.27
027	gi|425194	Heat shock protein [*Spinacia oleracea*]	112	25	70.8/5.15	68.6/5.50
028	gi|212276328	Hypothetical protein LOC100191878 [*Zea mays*]	82	20	59.5/9.72	55.3/6.18
029	gi|224174082	4-Coumarate-coa ligase [*Populus trichocarpa*]	86	14	16.6/9.03	12.3/5.78
030	gi|255618262	Conserved hypothetical protein [*Ricinus communis*]	82	12	20.3/11.86	22.3/6.16
031	gi|116055419	Unnamed protein product [*Ostreococcus tauri*]	89	22	52.5/9.06	50.7/5.96
032	gi|115486767	Os11g0701800	87	11	33.9/9.33	35.1/6.21
033	gi|79325139	Glycine-rich protein [*Arabidopsis thaliana*]	77	16	60.5/5.28	72.2/5.33

SC: spot coverage.
